# Exploring consumers’ perceptions of online purchase decision factors: electroencephalography and eye-tracking evidence

**DOI:** 10.3389/fnhum.2024.1411685

**Published:** 2024-11-18

**Authors:** Michal Pšurný, Stanislav Mokrý, Jana Stavkova

**Affiliations:** Department of Marketing and Trade, Faculty of Business and Economics, Mendel University in Brno, Brno, Czechia

**Keywords:** utilitarian and hedonic consumption, EEG and consumer behavior, online purchase behavior, event-related potentials (ERP), eye-tracing integration, consumer attention, consumer affective reaction

## Abstract

**Introduction:**

Consumer behavior on the Internet is influenced by factors that can affect consumers’ perceptions and attention to products. Understanding these processes at the neurobiological level can help to understand consumers’ implicit responses to marketing stimuli. The objective of this study is to use electroencephalography (EEG) to investigate the differential effects of selected online purchase decision factors that are becoming increasingly important in online shopping.

**Methods:**

Using event-related potentials (ERPs) and simultaneous eye-tracking measurements, we identified differences in the perception of utilitarian and hedonic products when the products are exposed together with visual elements of the factors review, discount, and quantity discount. The ERP analysis focused on the P200 and late positive potential components (LPP).

**Results:**

By allowing free-viewing of stimuli during measurement, early automatic and later more complex attentional affective responses could be observed. The results suggest that the review and discount factors are processed faster than the product itself. However, the eye-tracking data indicate that the brain processes the factor without looking at it directly, i.e., from a peripheral view.

**Discussion:**

The study also demonstrates the possibilities of using new objective methods based on neurobiology and how they can be applied, especially in areas where the use of neuroscience is still rare, yet so much needed to objectify consumers’ knowledge of their need satisfaction behavior.

## 1 Introduction

A multitude of factors influence consumer behavior. These factors are subject to change and evolution. The influence of some factors has diminished, while others have become less pronounced or even ceased to exist. New factors have emerged that are influencing consumer behavior in novel ways. The advent of the Internet and the possibility of online shopping have further amplified and accelerated this phenomenon (Gu et al., 2021; Prasetyo et al., 2021; Eger et al., 2021). Between 2011 and 2019, there was a notable increase in the proportion of Internet users in the V4 countries (Central Eastern European countries comprising Poland, the Czech Republic, Slovakia, and Hungary) who made online purchases of goods and services (Kral and Fedorko, 2021). The advent of the pandemic has precipitated a shift in consumer behavior, with quality, affordability, and convenience becoming paramount (Eger et al., 2021). This aligns with findings from other studies that have identified these factors as crucial, with price also emerging as a significant determinant alongside quality, availability, and convenience (Gu et al., 2021; Prasetyo et al., 2021).

Online reviews and discounting strategies are among the most significant factors influencing consumer behavior during online shopping, as evidenced by numerous studies (Fu et al., 2021; Niu et al., 2022; Chen D. et al., 2019; Chen W. et al., 2019; Zhang et al., 2010). A study conducted in Lithuania identified product price and customer reviews as the most important factors for shoppers, in addition to e-shop design and security, product design, and packaging (Davidaviciene et al., 2021). The results of a questionnaire survey conducted in 2015 in the Slovak Republic among 634 respondents indicate that “good reviews” are important to over 70% of online shoppers (Pollak and Dorčák, 2015). In comparison to the results of the same survey conducted in 2009, there has been an increase of over 15%. Furthermore, the 2015 survey indicated that 80% of respondents considered a good price to be an important factor.

In studying the factors that influence consumers on the Internet, it is essential to consider the distinction between utilitarian and hedonic consumption, as conceptualized in consumer psychology. This two-dimensional division is significant because it is based on disparate intrinsic consumer needs, and consumers exhibit disparate attitudes toward utilitarian and hedonic products (Dhar and Wertenbroch, 2000; Hu et al., 2020; Chen D. et al., 2019; Chen W. et al., 2019; Voss et al., 2003; Herbas Torrico et al., 2011). Utilitarian consumption is more functional, cognitively driven, goal-oriented, and practical. In contrast, hedonic consumption is focused on experience and excitement, is more emotion-driven, and is associated with pleasurable attributes (Herbas Torrico et al., 2011; Dhar and Wertenbroch, 2000; Voss et al., 2003). Consumers may have developed value systems that differ regarding utilitarian and hedonic consumption. For instance, Crespo-Almendros and Del Barrio-García (2016) corroborated the findings of Chandon et al. (2000), which indicated that consumers tend to favor incentives that offer utilitarian benefits for utilitarian products.

The objective of this study is to examine the impact and significance of diverse marketing factors utilized in the context of online shopping (online purchase decision factors). In light of the pivotal role discount and review factors play in determining the final purchase decision, marketing research is seeking to move beyond the confines of subjective opinion, which often manifests in the form of responses to questions. Consequently, there is a growing emphasis on identifying more objective methods to gain deeper insights. It is, thus, imperative to investigate the effect of these factors on consumers at an unconscious level using biometric and neuroscience methods. To obtain more objective results, electroencephalography (EEG) measurements are employed in conjunction with eye-tracking measurements. The EEG measurements focus on event-related potentials (ERP). The investigation of the latency and amplitude of ERPs, as measured by EEG, may facilitate the understanding of some of the sensory and cognitive processes related to consumption behavior elicited by experimental manipulation (McInnes et al., 2023). The measurement of ERPs remains an underexplored area of research, yet its importance is increasing. This is evidenced by the growing number of published papers in all disciplines (Donoghue and Voytek, 2022) and the growing aggregation of literature in the context of consumer behavior and marketing (McInnes et al., 2023; Hakim and Levy, 2019; Lin et al., 2018b). The use of EEG in marketing and consumer behavior research is of interest for several reasons. Primarily, it is a relatively inexpensive method of measuring voltage changes in milliseconds. Additionally, it can be deployed in real-world settings, such as a retail store (Berčík et al., 2021; Berčík et al., 2016). The study takes into account that consumption can be divided into utilitarian and hedonic and therefore asks whether:

Do factors important to online purchase decision-making influence the perception of products?

Is there a difference in the perception if they are utilitarian or, on the contrary, hedonic products?

An understanding of the disparate perceptions of these factors can assist retailers and e-shop owners in the development of novel marketing strategies. The present study focused on the influence of discount and review factors in conjunction with utilitarian and hedonic products. The aforementioned methods can be employed to elucidate discrepancies in affective perception—early automated or late emotional attention.

In the context of sensory stimuli, a distinct P200 component is often observed in the frontal, central, and parietal regions of the head, with the frontocentral region being the most commonly affected (Chen D. et al., 2019; Ma et al., 2018; Stahl et al., 2010; Li et al., 2005; Yuan et al., 2007). The P200 has been the subject of investigation in relation to a number of different phenomena, including language, selective attention (e.g., in the context of threat perception), and working memory. Furthermore, it has been the subject of investigation in a number of other paradigms (Lin et al., 2018b). A review article by Donoghue and Voytek (2022) indicates that the P200 wave is most frequently studied in the context of auditory perception, memory, visual perception, and attention.

The P200 component reaches a maximum of approximately 180–250 ms following stimulus onset in the context of visual perception and 170–180 ms in the case of auditory perception (Kotchoubey, 2006). The observation that the P200 component to auditory perception can be entirely eliminated when attention is directed toward visual perception indicates that the P200 is subject to modulation by attention, that is, selective attention (Michie et al., 1993). The P200 component has been employed in numerous studies as an indicator of early automatic attention. In other words, the P200 has been utilized as an indicator of attention-modulated perceptual processing (Jones et al., 2012; Lijffijt et al., 2009). In some cases, it has been linked to memory and recognition processes (Lijffijt et al., 2009; McEvoy et al., 2001). The study by Lijffijt et al. (2009) indicates a correlation between the amplitude of the P200 and working memory. Conversely, the latency of the P200 may be associated with attention. A reduction in P200 amplitude may be indicative of a rapid process of detecting stimulus-related features and content that attract attention automatically and rapidly, such as threatening content (Yuan et al., 2007; Li et al., 2005). A study by Jin et al. (2018) demonstrated that when products are presented with an eco-label, the P200 amplitude is significantly reduced. This indicates that the diminished amplitude may be associated with both the automatic processing of the eco-label and a favorable response to the stimulus.

Nevertheless, the activation of neural processes associated with attention is closely linked to other psychological processes, such as affective reactions (emotional responses). Similarly, the relationship between the P200 component and attentional processes has been previously described in the context of emotionally attuned content. The P200 may be the initial component for responding to affective valence (Delplanque et al., 2004). Such a response may be associated with an expeditious emotional response to a stimulus before cognitive processing (Ma et al., 2018). The amplitude of this component was observed to be higher in the frontal and central regions of the scalp in studies conducted by Delplanque et al. (2004) and Carretié et al. (2001) in response to negatively arousing stimuli. In the study conducted by Wang et al. (2012), a markedly diminished P200 amplitude was observed when positive sensation-eliciting stimuli were presented. In the study conducted by Jin et al. (2017), messages with negative framing elicited a larger P200 amplitude. It can be reasonably inferred that negative messages are likely to attract a greater allocation of attentional resources, thereby eliciting larger P200 amplitudes (Jin et al., 2017). The P200 is indicative of the automatic mobilization of attentional resources to negative stimuli (Wang et al., 2012), which may be related to the brain’s response to a potential threat that is of greater importance than a positive stimulus in terms of survival (Yuan et al., 2007; Li et al., 2005). In the study conducted by Carretié et al. (2001), the amplitude was observed to be higher and the latency shorter in response to negative stimuli than positive stimuli. However, while no correlation was observed between latency and the valence content of the stimulus, a significant relationship was identified between the amplitude at the frontal and central regions and the valence content of the stimulus. The shorter P200 latency was observed for stimuli with higher emotional content, which may reflect a reflexive attentional direction to hedonic stimuli (Schupp et al., 2004; Junghöfer et al., 2001). The aforementioned findings indicate a close association between emotion-related activity and attentional activity, suggesting that the two processes are intimately connected. In a natural setting, attention is maintained by stimuli with emotional significance, particularly negative stimuli that pose a threat to survival, as opposed to routine and neutral stimuli (Carretié et al., 2001). The rapid processing of emotionally salient content may be associated with the subsequent, more complex processing of emotional content, as reflected in the P300 and late positive potential (LPP) components, where longer latencies of these components are observed (Schupp et al., 2004).

In particular, the LPP component has been demonstrated to be associated with affective perception. The reliability of this component as an indicator of subsequent emotional processing has been demonstrated in multiple studies (Mastria et al., 2017; Pozharliev et al., 2015; Minnix et al., 2013; Schupp et al., 2004; Sabatinelli et al., 2006; Hajcak et al., 2006; Lin et al., 2018a). The LPP is a wave that occurs in the parietal or occasionally centro-parietal head region within 400–800 ms of stimulus onset (Chen D. et al., 2019; Chen W. et al., 2019). The peak typically occurs around 600 ms (Zhang et al., 2019; Herring et al., 2011; Sabatinelli et al., 2006). In several studies, a higher LPP amplitude was observed when participants were presented with both pleasant and unpleasant images of high emotional intensity compared to neutral images (Mastria et al., 2017; Minnix et al., 2013; Sabatinelli et al., 2006; Hajcak et al., 2006). The results demonstrated a more favorable outcome when participants were permitted to free-view the images without engaging in any behavioral tasks. The presentation of the same images on multiple occasions had only a moderate effect on the size of the LPP. This suggests that the cortico-limbic motivational systems are engaged automatically (Mastria et al., 2017). In light of these findings, it can be proposed that the LPP is associated with the involvement of brain motivational circuits and reflects the emotional arousal associated with emotionally motivated attention (Sabatinelli et al., 2006; Pozharliev et al., 2015). Hajcak et al. (2010) put forth the proposition that the LPP can be employed as a means of quantifying emotional reactivity and regulation. The amplitude of the LPP is determined by the willful modulation of emotions, reappraisal instructions, preceding descriptions, the way in which stimuli are initially appraised, and manipulations of attention. In contrast with the findings of Jin et al. (2017), which indicated that messages with negative framing elicited a larger amplitude of the P200, the present study demonstrated that positive framing elicited a larger amplitude of the LPP. Furthermore, the LPP amplitude was found to be correlated with the purchase intentions of the study participants.

In their studies of utilitarian and hedonic consumption, Hu et al. (2020), Chen D. et al. (2019), and Chen W. et al. (2019) employed the ERP method. Specifically, Hu et al. (2020) investigated the impact of sexually oriented advertisements on utilitarian or hedonic products by examining the N200 and LFSW components. The results demonstrated that advertisements with a sexual motivation are more effective for hedonistic products. Chen D. et al. (2019) and Chen W. et al. (2019) conducted a comparative analysis of the P200, P300, and LPP components of hedonistic and utilitarian labels between high and low-social status groups. The results demonstrated that the low-social-status group exhibited a markedly diminished P200 amplitude in response to hedonic labels compared to utilitarian labels. Furthermore, the latency of this component was shorter for hedonic labels in individuals with low social status. This indicates that individuals with low social status perceive hedonic labels as a threatening content. The LPP analysis revealed a dynamic trend for individuals with low social status in the case of hedonic labels, suggesting that they elicit negative emotions. Consequently, the low-social-status group is more susceptible to hedonistic product information because it evokes a sense of threat and associated negative emotions.

It is not a common practice in the field of ERP studies to incorporate simultaneous eye-tracking measurements into investigations focusing on marketing and consumer behavior. As a result, it is often difficult to ascertain with precision the exact location to which a participant directs their visual attention. Recently, however, studies combining these methods have begun to emerge (Kulke et al., 2021). Moreover, the role of peripheral vision in visual stimulation merits consideration. The term “peripheral vision” refers to the visual field extending beyond the central gaze, enabling the reorientation of attention (Keshvari and Rosenholtz, 2016). Kulke et al. (2021) conducted a study combining EEG with eye-tracking and concluded that the emotional content of the stimulus (face) amplifies early automatic neural responses, irrespective of whether attention is accompanied by eye movements.

## 2 Materials and methods

The study design comprised a pre-test (December 2021) and experimental measurements (EEG and eye-tracking measurements, May 2022). Based on the pre-test, utilitarian, hedonic products, and online purchase decision factors were selected and displayed to participants in the experimental measurements.

### 2.1 Stimulus design pre-test

The pre-test, which used a questionnaire survey distributed online via an Umbrella app^1^, was completed by 489 respondents (mean age = 22.78; SD = 5.54). The respondents rated 30 randomly selected product images with respect to some products from the study conducted by Hu et al. (2020). The respondents evaluated the type of the product using a 7-point scale, with 1 indicating the most utilitarian and 7 indicating the most hedonic. They were presented with a definition from the Hu et al. (2020) study:

“Hedonic products focus on feelings and experiences and are fun, exciting, and enjoyable. Utilitarian products focus on functionality and utility and are effective, beneficial, functional, and practical. Utilitarian products highlight their functional and practical utilities.”

A total of 30 product images consisted of *Digital alarm clock, Flash disk (32GB), Teapot, Spice Grinder, French Press, Electric Kettle, Memory Card (32GB), Shaker, Wired Headphones, Wireless Doorbell, Tea Service, Mocca Teapot, Analog Alarm Clock, wireless headphones, condoms, vitamin tablets, shampoo, plush toy, perfume, massage oil, music album, energy drink, honey cake, photo album, vibrator, diapers, sweatshirt (unisex), bracelet, hiking boots, and erotic inflatable doll*.

The individual responses of the respondents on a 7-point scale were transformed into the form of means. The product type was verified for five products that scored close to one (utilitarian product type) and five products that scored close to seven (hedonic product type). An exact *t*-test with one-sample mean was employed for verification, with *μ* = 3.5 corresponding to the mean of the score scale. For both product types, the null hypothesis was stated as follows: the sample mean does not differ from the mean (μ = 3.5). The rejection of the null hypothesis thus demonstrates the relevant product type (utilitarian and hedonic).

The randomly selected online purchase decision factors from the Czech e-shop Alza.cz^2^ were evaluated by respondents on a 5-point scale. These factors included as follows: the sale price, discount information, reviews, quantity discount, number of reviews, number of recommending customers, return option (60 days), number of last units, number of customers who have purchased, low complaint rate, information indicating that the product can be purchased on the e-shop, quantity discount if more agreed customers buy, and information on the number of stores where the product is displayed. Based on the frequency of responses, the review, discount, and quantity discount were selected.

**Figure 1:**
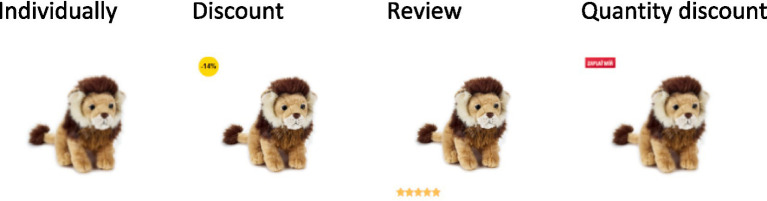
An example of different variants of visual stimuli for the selected product (plush toy).

In the experimental measurements, the final 10 products (five utilitarian and five hedonic) were displayed in four variants: a product without a factor (individually), a product with a discount factor, a product with a review factor, and a product with a quantity discount factor. A sample variation for one of the products provided to participants is shown in Figure 1.

### 2.2 Experimental measurements

The experimental measurements were conducted with the participation of 32 volunteers, selected from among students at Mendel University in Brno, Czech Republic (16 women, 16 men, mean age = 23.36; SD = 2.29). Of note, 31 volunteers (participants) were right-handed, and one was left-handed. All participants were queried about ocular and neurological conditions. Lenses were permitted. If the subject exhibited astigmatism, no measurement was taken. The experimental measurements were approved by the Ethics Committee at Mendel University in Brno, and all participants signed an Informed Consent Form.

We used the methodology described by Telpaz et al. (2015), which was also utilized by Hu et al. (2020) in their research. The experiment was thus divided into the following three phases: (1) familiarization with the products and online purchase decision factors before the measurement; (2) EEG measurement, wherein the products were presented to the participant; and (3) finally, the participant rated the products in a questionnaire (behavioral task).

The experiment was conducted at the ETLab, Faculty of Business and Economics, Mendel University.^3^ Upon coming to the laboratory, the researcher explained the experimental procedure comprehensively to the participant. The participant then signed the Informed Consent Form, was shown the 10 selected products, and was given an explanation of the infographics depicting online purchase decision factors—discount, quantity discount, and review (more positive reviews). Subsequently, the participant was fitted with an EEG cap and seated in front of a monitor screen, upon which the stimuli were subsequently displayed. The participant was instructed to minimize movements and avoid clenching the neck muscles. Subsequently, the electrode resistances on the EEG cap were adjusted, and the eye tracker was calibrated.

**Figure 2:**
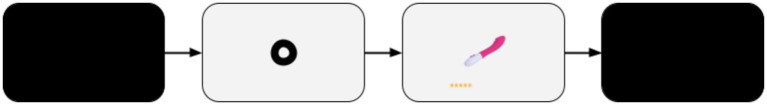
An example of a trial: blank screen, drift correction (1,000 ms), stimulus (2000 ms), and blank screen.

The experimental measurement was divided into five blocks, with 42 trials in each block, for a total of 210 trials. Trials were divided into a blank screen, drift correction (1,000 ms), stimulus (2000 ms), and a blank screen. The sequence in a single trial is shown in Figure 2. A short pause was allowed between blocks, during which the participant could rest. The participant then initiated the next block by pressing a button on the keyboard. To help the participant maintain focus, large and small circles were randomly displayed during the product presentation. When a large circle was displayed, the participant was instructed to press the keyboard button (space bar). The order of the products in the trials was randomized, but all products at all variants were presented the same number of times. Thus, for a single product, there were—5x products alone, 5x products with discount, 5x products with quantity discount, and 5x products with review. For 10 products, that’s 200 trials plus 5x small circles and 5x large circles. After the measurement was completed, the participant still had to fill out a short questionnaire. The participant rated the type of products (utilitarian/hedonic)—the same question as in the pre-test (7-point scale).

### 2.3 Technical aspects of measurement

For EEG recording, a LiveAmp 32 amplifier (Brain Products) was used in conjunction with an EEG cap 32 channel R-Net electrode system configuration and passive Ag/AgCl electrodes (Brain Products). An SR Research EyeLink^®^ 1,000 Plus eye-tracker was used to record eye movements. The stimulus sequence was created in the Experiment Builder software (SR Research) on a Stimulus PC, which was also used to display the stimuli during the experimental measurements. The Stimulus PC also stored the eye-tracking signal data. To synchronize the EEG and eye-tracking signals, a Trigger Box (Brain Products) was used to distribute the marker (trigger) at the moment of stimulus display on the Stimulus PC monitor to the EEG signal (LiveAmp 32 amplifier). The EEG data were stored on the Recording PC using the BrainVision Recorder software. A deviation of 0.5° was set as the calibration limit of the eye-tracker, and the right eye was scanned. The experiment started with an electrode resistance on the cap below 5 kΩ. The display area of the monitor was set to 1024 × 768 px, the color depth to 32 bits per pixel, and the refresh rate to 60 Hz. Each of the presented stimuli had the same size as 310 × 310 px, and the graphical representation of the review, discount, and quantity discounts was placed in the same coordinates as each presented stimulus. The sampling rate was set to 500 Hz on both the eye-tracking and EEG devices.

### 2.4 Eye-tracking data analysis

Three areas of interests (AOIs) were created to analyze the eye-tracking data: one for the review factor, one for the discount factor, and one for the quantity discount factor. According to the above specifications, the AOIs were defined to correspond to the area of the graphical representation of review, discount, and quantity discount. The observed AOIs were positioned in identical coordinates for the same factors for all ten products. The selected AOI metrics include: Time to first fixation (time to the start of the first fixation in the AOI in milliseconds relative to the start of the stimulus presentation), duration of first fixation in ms, and dwell time in the AOI in ms. Another metric presented is the average pupil size in arbitrary units (AUs), based on the number of pixels on the eye-tracking camera that are thresholded and considered part of the pupil. Eye-tracking AOI data were exported using Data Viewer (SR Research). It was then averaged across all measurements, and a summary table was created. The non-parametric Wilcoxon test was used to compare the metrics for each product factor (utilitarian/hedonic). The *p*-values for each factor were then summarized in the table. For example, for the discount—AOI discount (utilitarian) versus AOI discount (hedonic). The Wilcoxon signed-rank test was calculated from the original unaveraged scores. The IBM^®^ SPSS^®^ Statistics 25 software was used for the calculation.

### 2.5 EEG data analysis

The EEG data analysis was performed on a total of 30 participants. Two participants were excluded from the analysis, one due to the unavailability of a usable recording and the other due to their left-handedness. The online reference electrode FCz was referenced to electrodes P9 and P10, as a useful signal in the fronto-central region was assumed (Telpaz et al., 2015). In addition, the data were filtered with an IIR filter from 0.5 to 40 Hz. Independent component analysis (Hyvärinen and Oja, 2000; Jung et al., 1998) was used to remove the eye, muscle, and other artifacts. The data were segmented by utilitarian and hedonic products combined with products presented alone, with review, with discount, and with quantity discount. Segmentation was always performed within a time window of −200 to 800 ms from stimulus onset. Baseline correction was performed from −200 ms to stimulus onset. These segments were averaged per participant, followed by a grand average across all subjects.

Based on visual inspection of topographic maps and previous studies (Ma et al., 2018; Chen D. et al., 2019; Chen W. et al., 2019), electrodes Fz, F3, F4, Cz, C3, C4, P3, P4, and Pz were selected for deeper analysis of the P200 component, with a time window of 160–210 ms. These electrodes were then pooled, and the resulting signal was analyzed.

For LPP analysis, a time window of 500–700 ms was chosen (Ma et al., 2018; Pozharliev et al., 2015), and electrodes C3, C4, CP1, CP2, CP5, CP6, Cz, P3, P4, P7, P8, and Pz were selected. These electrodes were pooled for the final signal, similar to the P200 component.

ANOVA was used to test the effect of each factor separately for utilitarian and hedonic products, with the independent variable being the data from the respective ERP component time window (P200, 160–210 ms; LPP, 500–700 ms). EEG data analysis was performed using the BrainVision Analyzer 2 software. The exported ERP data were analyzed using the IBM^®^ SPSS^®^ Statistics 25 software.

### 2.6 Validation of products for the experiment

Subsequently, after the experiment, it was necessary to determine the degree of agreement between the participants’ perceptions and the respondents in the pre-test regarding the product type (utilitarian, hedonic). The participants were asked the same questions as the respondents in the pre-test. The level of agreement was evaluated by conducting a one-sample *t*-test, where the sample mean was calculated based on the seven-point scale values of the utilitarian and hedonic products previously identified as utilitarian and hedonic in the pre-test. The value *μ* = 3.5 was taken as the mean of the scale scores. The null hypothesis was stated in the same way for both product types: the sample mean is not different from the mean. The rejection of the null hypothesis indicates the existence of a consensus regarding the nature of the products in question (utilitarian, hedonic).

Participants in the experiment indicated their preferences for each product on a 7-point scale (1—least preferred; 7—most preferred). The preference scores for each product in the experiment were obtained by transforming the participants’ scores using the mean. The preference scores for utilitarian and hedonic products were tested with a two-sample *t*-test, with the null hypothesis that preferences for utilitarian and hedonic products are identical.

## 3 Results

### 3.1 Stimulus design pre-test

Table 1 shows the respondents’ mean scores for the selected utilitarian and hedonic products in the pre-test, along with their corresponding characteristics. A one-sample *t*-test (*t* = 24.09; *p* < 0.001) confirmed that the digital alarm clock, flash disk, electric kettle, memory card, and diapers were perceived by respondents in pre-test as utilitarian products. The plush toy, music album, honey cake, vibrator, and erotic inflatable doll were demonstrably perceived as hedonic products (*t* = 27.13; *p* < 0.001). Therefore, these products were selected for experimental measurement.

**Table 1:** Characteristics of the chosen products of respondents in the stimulus design pre-test and participants in the experimental measurements.

Chosen products		Product character SD pre-test		Product character experiment		Preference score experiment	
		*x̄*	SD	*x̄*	SD	*x̄*	SD
Utilitarian	Digital alarm clock	1.82	1.33	1.50	0.67	3.08	1.78
	Flash disk	1.83	1.14	1.42	0.67	4.25	1.60
	Electric kettle	1.96	1.31	2.25	1.14	4.08	1.73
	Memory card	2.10	1.49	2.25	0.45	3.58	1.83
	Diapers	1.70	1.29	1.67	1.15	1.83	1.98
Hedonic	Plush toy	5.73	1.48	5.67	0.58	2.92	2.09
	Music album	5.82	1.49	6.83	1.04	3.25	0.89
	Honey cake	5.56	1.67	6.00	0.67	6.33	1.98
	Vibrator	6.05	1.49	6.50	0.65	2.92	0.94
	Erotic inflatable doll	5.94	1.62	6.50	0.80	1.75	1.22

### 3.2 Validation of products for the experiment

To ensure that the experiment participants perceived the product type in a manner consistent with the pre-test respondents, a questionnaire was administered after the experimental measurement. This questionnaire asked about the utilitarian or hedonic type of the products. Table 1 shows the means of the experimental participants’ ratings of the product types. The results of a one-sample mean *t*-test corroborate the hypothesis that the experiment participants perceived both the utilitarian and hedonic types of the products similar to the pre-test respondents.

The preferences of the experiment participants toward the presented products can influence the trajectories of ERP components (Goto et al., 2019; Telpaz et al., 2015). Accordingly, experiment participants were asked about their preferences for each product. Subsequently, the preference scores for each product were determined by transforming them using the means, as shown in Table 1. The results of the preference scores for utilitarian and hedonic products, as well as the result of a two-sample *t*-test (*t* = 0.07; *p* = 0.94), indicate that the null hypothesis of preference for the type of product is not rejected. This suggests that the preference scores for utilitarian and hedonic products are not statistically different.

### 3.3 Eye-tracking results

Eye-tracking data from selected AOIs are used to compare visual attention. In addition, average pupil size data from the aforementioned AOIs are presented. Table 2 provides a comparison of data for the review, discount, and quantity discount incentives across selected metrics for utilitarian and hedonic products.

**Table 2:** Average values of eye-tracking metrics concerning selected AOI.

	Utilitarian			Hedonic		
	Review	Discount	Quantity discount	Review	Discount	Quantity discount
AOI time to first fixation (ms)	1928.25	1758.07	1543.00	1748.00	1562.00	1534.00
AOI duration of first fixation (ms)	422.55	437.61	422.00	410.00	402.00	496.00
AOI dwell time (ms)	473.22	528.07	509.00	530.00	476.00	596.00
AOI average pupil size (AU)	367.64	349.40	342.00	340.00	367.00	323.00

#### 3.3.1 Time to first fixation

In the case of utilitarian products, quantity discount emerged as the most salient factor in terms of speed of attention. For hedonic products, quantity discount was also the most influential factor, although the time required was not significantly different from the other factors. The review factor had the longest reaction time compared to the other incentive levels for both product types. The review and discount factors showed a shorter time to the first fixation for utilitarian products. A Wilcoxon test yielded a statistically significant result, indicating that the discount was perceived as more attractive for utilitarian products (*p* = 0.047). The time to first fixation was found to be significantly longer for the discount and review factors for utilitarian products.

#### 3.3.2 Duration of first fixation

The quantity discount was found to have a significantly longer duration of first fixation for utilitarian products than the discount and review factors. All factors had a relatively similar duration of first fixation for utilitarian products. However, the discount and review factors had a significantly shorter duration for hedonic products than utilitarian products (discount, Wilcoxon, *p* = 0.012; review, Wilcoxon, *p* = 0.032).

#### 3.3.3 Dwell time in AOI

Table 2 shows that the dwell time in AOI is longer for the quantity discount and review in the case of hedonic products. For discount in the case of utilitarian products. The Wilcoxon test confirmed that the times were significantly different for discount (Wilcoxon; *p* < 0.001) and review (Wilcoxon; *p* = 0.019) when comparing factor versus utilitarian/hedonic products.

#### 3.3.4 Average pupil size

Pupil size was significantly different for discount (Wilcoxon; *p* = 0.050) when comparing utilitarian versus hedonic. Table 3 summarizes the *p*-values of the Wilcoxon test for all the stimuli.

**Table 3:** P-values of the Wilcoxon test.

	Review	Discount	Quantity Discount
Time to first fixation	0.060	0.047	0.525
Duration of first fixation	0.032	0.012	0.707
Dwell time in AOI	0.019	0.000	0.052
Pupil size	0.984	0.050	0.684

The *p*-values for individual stimuli in comparison with utilitarian versus hedonic. Significant differences are marked in red.

### 3.4 EEG results

**Figure 3:**
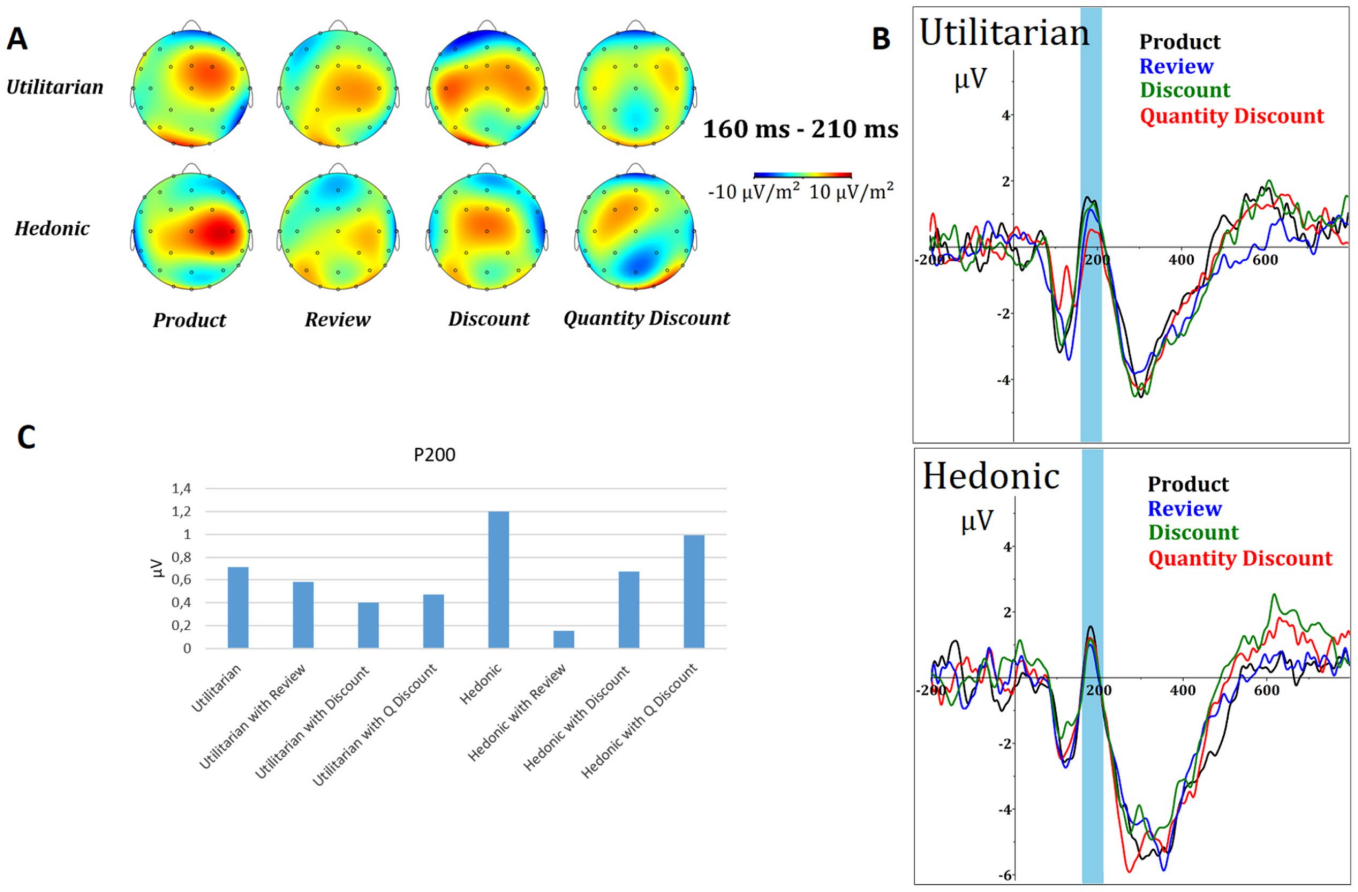
P200 results. **(A)** Topographic map of voltage (μV) for all electrodes in the time window 160–210 ms. **(B)** Courses of the ERP component (pooling electrodes Fz, F3, F4, Cz, C3, C4, P3, P4, and Pz) of utilitarian (top) and hedonic (bottom) products for all combinations (without a factor, review factor, discount factor, and quantity discount factor). **(C)** Mean voltage (μV) amplitude of P200 for all combinations.

The P200 component was chosen to elucidate participants’ early initial attentional processing across all variants of visual stimuli, including both with and without online purchase decision factors. Figure 3 shows the scalp voltages at 160–210 ms (A) for all combinations. The first column, named product, means the scalp voltages without the online purchase decision factor, i.e., only the product itself. The ERP component waveform (B) is shown for utilitarian (up) and hedonic (down) products.

The ERP waveforms indicate that the P200 amplitudes for both utilitarian and hedonic products are reduced when the product is presented with the online purchase decision factor. In addition, the P200 wave for utilitarian products has a longer overall latency. The results of the ANOVA indicated that the online purchase decision factors had a significant effect on utilitarian products (*F* = 32.404; *p* < 0.001). The most significant changes were observed in response to the quantity discount (*p* < 0.001) and review (*p* < 0.001). The effect of the discount was not significant (*p* = 0.271). The application of factors did not have a discernible effect on hedonic products (*F* = 1.202; *p* = 0.313). The results for the online purchase decision factors of review, discount, and quantity discount yielded *p*-values of 0.228, 0.535, and 0.886, respectively.

**Figure 4:**
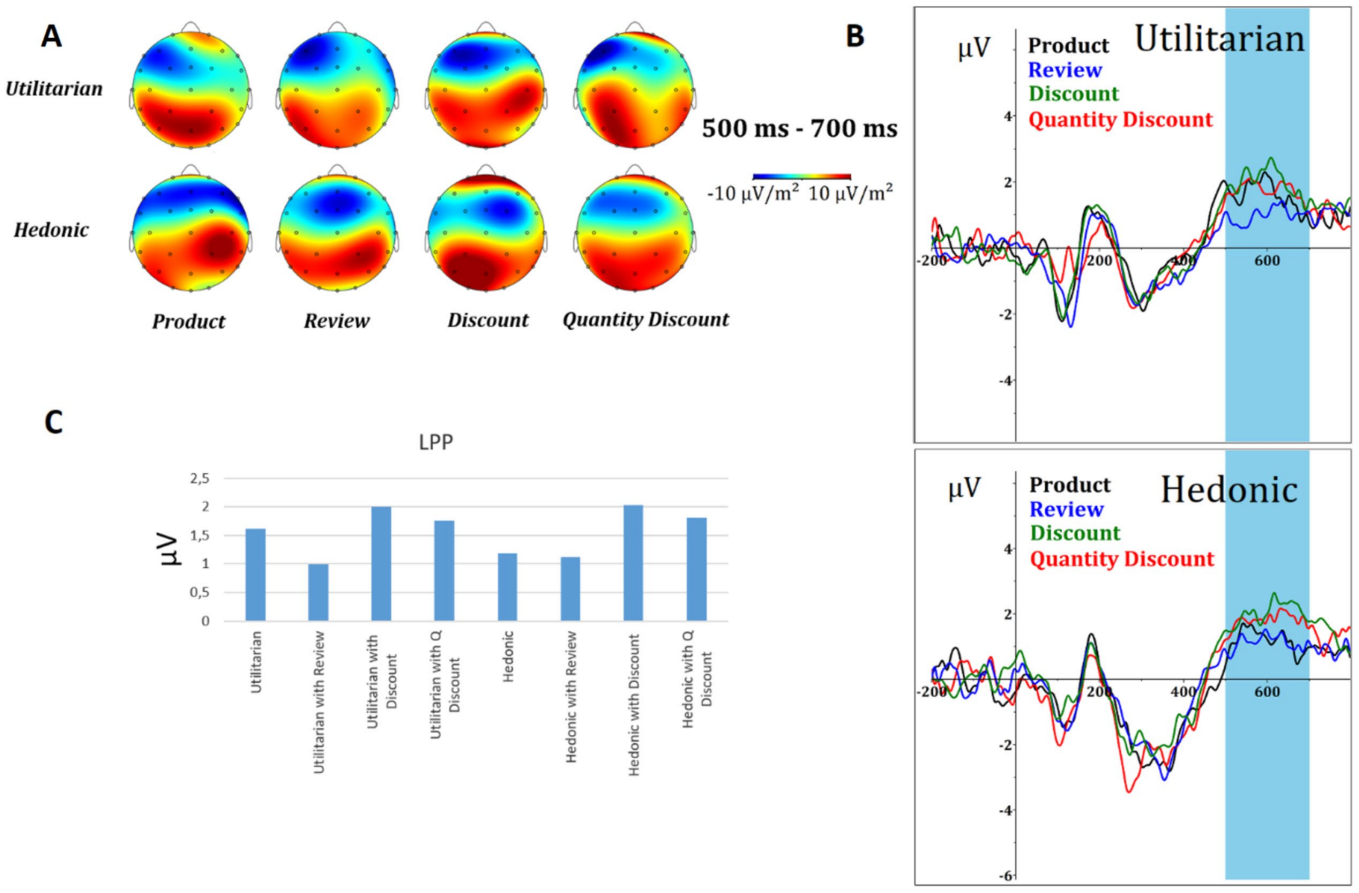
LPP results. **(A)** Topographic map of voltage (μV) for all electrodes in the time window 500–700 ms. **(B)** The courses of the ERP component (pooling electrodes C3, C4, CP1, CP2, CP5, CP6, Cz, P3, P4, P7, P8, and Pz) of utilitarian (top) and hedonic (bottom) products for all combinations (without a factor, review factor, discount factor, and discount quantity factor). **(C)** Mean voltage (μV) amplitude of LPP for all combinations.

The LPP component was defined by a time window of 500–700 ms. The topographic maps (A) for utilitarian and hedonic products presented either alone or in combination with the online purchase decision factor are shown in Figure 4, together with the ERP waveform (B). The results of the ANOVA demonstrated the impact of the factors on the LPP component waveform for both utilitarian (*F* = 168.866; *p* < 0.001) and hedonic (*F* = 258.554; *p* < 0.001) products. In the case of utilitarian products, all stimulus levels were found to be significant: review (*p* < 0.001), discount (*p* < 0.001), and quantity discount (*p* = 0.011). In contrast, only discount (*p* < 0.001) and quantity discount (*p* < 0.001) were found to be significant in the case of hedonic products. For the review, *p* = 0.407.

## 4 Discussion

The objective of this study was to examine the significance of factors influencing online shopping behavior (Fu et al., 2021; Niu et al., 2022; Chen D. et al., 2019; Chen W. et al., 2019; Zhang et al., 2010). The study examined the perceptions of the online purchase decision factors (reviews, discounts, and quantity discounts) in combination with utilitarian and hedonic products. ERPs measurement was employed to examine the P200 and LPP. To elucidate the discrepancies in visual attention, eye-tracking metrics time to first fixation, duration of first fixation, and dwell time in AOI were presented, as well as pupil size data. The time to first fixation indicates the speed with which the factor gained attention, while duration of first fixation indicates the level of engagement the factor gained when first looked at. The dwell time in AOI indicates the duration of not only the first fixation but all subsequent fixations.

The study employed the methodology proposed by Plassmann et al. (2015), with a particular focus on identifying the underlying mechanisms and measuring implicit processes to assess early attention, visual attention, and affective perception. This study presents a measurement of ERPs using an EEG device simultaneously with an eye tracker device, a relatively uncommon practice in the field. The simultaneous measurement of eye movements and ERPs has yielded intriguing results. Given the nature of free-viewing images, the study focused specifically on attention and affective perception at the level of automatic processing and the involvement of cortico-limbic motivational systems (Mastria et al., 2017; Pozharliev et al., 2015). A number of studies have demonstrated a relationship between the waveform of P200 and LPP components in the context of emotionally evocative images. However, these studies have primarily focused on images that elicited survival-related importance to participants, such as depictions of death, threat, and violence. The question of whether these findings can be generalized to items of economic value, such as consumer goods, remains unresolved. While there have been recent developments in this area, the number of studies is still limited (Goto et al., 2017).

The results of this study demonstrated that at 160 ms (160–210 ms) following the onset of the stimulus, a notable P200 component was observed, indicative of a response to visual stimuli (Kotchoubey, 2006). The P200 amplitude was observed to be smaller for both utilitarian and hedonic products when presented with the discount and review factor. This suggests that participants initially processed the discount or review factor information and subsequently focused on the product. A reduction in amplitude can indicate an expedited processing of the stimulus-related feature (Yuan et al., 2007). In a study conducted by Lijffijt et al. (2009), the authors posit that the magnitude of the amplitude may be related to the working memory load. These findings are consistent with the assumption that the factor does not place as great a load on working memory as the product itself. In the study by Jin et al. (2018), the amplitude was significantly smaller when the product was presented with an eco-label. The present study reaches essentially similar conclusions. The amplitude is observed to be smaller when the product is presented with a graphical representation of the factor with a certain function. Jin et al. (2018) posited that the processing of the eco-label may be both automatic and elicit a positive response. However, data from the AOI obtained from eye-tracking (time to first fixation always >1.5 s) indicate that participants looked at the factor significantly later than the P200 component was induced. It can thus be assumed that the response is not related to the direct gaze in the graphical representation of the factor. Factors were processed by attention using peripheral vision, which is consistent with the study by Kulke et al. (2021), where neural responses to emotional stimuli were also not accompanied by eye movements. This area could be the subject of further research.

The concept of attention is inextricably linked to the affective response to a stimulus. Indeed, numerous studies have elucidated the relationship between the P200 component and emotionally attuned content. The occurrence of shorter latencies has been documented in the context of emotionally attuned content (Schupp et al., 2004; Junghöfer et al., 2001). This is in accordance with the findings of the present study. A shorter latency was observed for hedonic products, both with and without additional factors, than utilitarian products. This is consistent with the findings of Herbas Torrico et al. (2011), who observed that utilitarian consumption is more driven by cognitive processes, whereas hedonic consumption is driven by affective processes (emotional and immediate). The reduction in amplitudes for the products presented with the online purchase decision factors may be interpreted as an indication of a more positive response to the stimulus. This is based on the observation that lower P200 amplitudes were associated with positive stimuli, whereas negative stimuli exhibited higher amplitudes (Wang et al., 2012; Delplanque et al., 2004; Carretié et al., 2001). In the study conducted by Carretié et al. (2001), the amplitude was also found to be valence-related. It can thus be inferred that a lower amplitude indicates a more positive response to the stimulus. The notable alteration in the P200 waveform in response to the quantity discount for utilitarian products aligns with the conclusions of Crespo-Almendros and Del Barrio-García (2016) and Chandon et al. (2000). These researchers have established that consumers tend to favor incentives offering utilitarian benefits (that the quantity discount exemplifies). This finding is also corroborated by the results of the eye-tracking analysis. The time required for the initial fixation was shortest for the quantity discount presented with utilitarian products, indicating that this online purchase decision factor received the fastest attention compared to the other factors.

Another ERP component that was observed and analyzed was the LPP component, which occurred between 500 and 700 ms. The LPP component exhibits a diminished voltage waveform when utilitarian products are presented with the review factor. In the case of discount factors, the waveform is analogous to that observed for the product presented alone. In contrast, the review factor exhibited a similar waveform for utilitarian products when presented alone and a higher waveform for discount and quantity discount. The results of numerous studies indicate that a higher pass-through occurs when emotionally attuned content is presented than neutral content (Pozharliev et al., 2015; Minnix et al., 2013; Yen et al., 2010; Schupp et al., 2004; Sabatinelli et al., 2006). It can thus be inferred that reviews reduce emotional perception in the case of utilitarian products. It may be assumed that reviews induce a sense of security and elicit a lower emotional response. In contrast, discounts were shown to elicit an emotional response more frequently in the case of hedonic products. The discount was demonstrated to cause a larger pupil size for hedonic products, which could also indicate an affective response.

Time to first fixation shows that participants noticed quantity discounting the fastest for utilitarian products compared to review and discount factors. Reviews and discounts were noticed faster for utilitarian products than for hedonic products. In the case of hedonic products, duration of first fixation and dwell time in AOI are longer for quantity discount than review and discount. This suggests that participants thought more about the meaning of quantity discounting for hedonic products and what the benefit (benefit of more of the same product) would be for them. Duration of first fixation and dwell time in AOI are significantly shorter for quantity discounts, specifically for utilitarian products, than hedonic products. This also indicates automated attention—participants did not spend as much time understanding the incentive. The longer time to first fixation for discount and review than quantity discount in the case of utilitarian products suggests that this may not be as important an attribute for utilitarian products. Time to first fixation may also have been influenced by the complexity of the product images presented. The importance of the quantity discount incentive is also illustrated by the average pupil size, which is also larger for quantity discount in the case of utilitarian products than hedonic products.

### 4.1 Limitations

This study represented a free-viewing task—participants were instructed to observe the presented stimuli and reflect on the value of the products, as was the case in previous studies (Telpaz et al., 2015; Hu et al., 2020). Therefore, no behavioral tasks were performed (Goto et al., 2017), and the Oddball paradigm was not employed (Chen D. et al., 2019; Chen W. et al., 2019). This should not be a problem because Mastria et al. (2017) demonstrated that LPP modulation is not significantly influenced if the images are presented freely or if the images are presented during a categorization task. The sample of participants for the experiment corresponded to Generation Z, with a mean age of 23.36 years (SD = 2.29 years). This selection was based on the sample from the questionnaire survey. It is pertinent to question whether the data would have yielded different results had the measurement been conducted in a different age group. However, fundamental principles of brain physiology that are common to consumers do not undergo as many changes as other behavioral variables (Lin et al., 2018b). Nevertheless, reaction time does slow with age, and slight changes can then be observed during the P200 component (McEvoy et al., 2001).

In the case of measuring with an eye camera, the data could affect the complexity of the presented product images. This refers to the number of lines and colors present in the product image, among other characteristics. The complexity of the image is undoubtedly a contributing factor in determining the number of fixations. The products included two erotic aids as follows: a vibrator and an erotic inflatable doll. Given that the participants were divided equally between female and male, it can be reasonably assumed that this product category will not exert a significant influence on the overall evaluation of the product by the participants. Participants demonstrated a clear preference for the honey cake product over the other products. Concerning preferences, it is possible that the honey cake product exerted a greater influence on participants, given that it is the only product intended for immediate consumption. The results of the ANOVA demonstrated a statistically significant alteration in LPP waves for all stimulus combinations of both utilitarian and hedonic products. The significant change was primarily attributable to the distinct waveforms of the LPP component, which exhibited less smoothness in this instance. The study investigated consumer perception of the aforementioned online purchase decision factors and solely examined early automatic attention and affective responses to the factors and products. However, it did not report on positivity, negativity, or the effect on sales performance. The study exclusively focused on positive reviews. A follow-up study could, for instance, concentrate solely on reviews and do so in the context of positive and negative reviews (vs. neutral stimuli) and utilize the so-called Oddball paradigm.

## 5 Conclusion

This study examined the influence of discount, quantity discount, and review on consumer perceptions of demonstrable utilitarian and hedonic products. Early automatic visual attention and affective response were quantified using the ERP method—P200 and the LPP component analysis. The presentation of products with factors reduced P200 amplitude in all cases. The shorter latency observed in the case of hedonic products indicates that these products are subjected to more affective processing. The ERP measurement results indicate that discount and review factors are processed more rapidly than the products themselves. The eye-tracking results demonstrate that these brain responses are not associated with direct gaze on the factor, but rather, the information is processed by peripheral vision. The LPP results illustrate that discounts enhance the subsequent emotional response in the context of hedonic products. Conversely, reviews diminish the subsequent emotional response in the case of utilitarian products, which may be attributed to feelings of certainty.

## Data Availability

The raw data supporting the conclusions of this article will be made available by the authors, without undue reservation.

## Footnotes

^1^https://umbrela.mendelu.cz/

^2^https://www.alza.cz/

^3^https://etlab.cz/
